# Dietary Heme Alters Microbiota and Mucosa of Mouse Colon without Functional Changes in Host-Microbe Cross-Talk

**DOI:** 10.1371/journal.pone.0049868

**Published:** 2012-12-11

**Authors:** Noortje IJssennagger, Muriel Derrien, Gerdien M. van Doorn, Anneke Rijnierse, Bartholomeus van den Bogert, Michael Müller, Jan Dekker, Michiel Kleerebezem, Roelof van der Meer

**Affiliations:** 1 Top Institute Food and Nutrition, Wageningen, The Netherlands; 2 Nutrition, Metabolism & Genomics group, Division of Human Nutrition, Wageningen University, The Netherlands; 3 Laboratory of Microbiology, Wageningen University, The Netherlands; 4 NIZO Food Research, Ede, The Netherlands; National Institute of Agronomic Research, France

## Abstract

Colon cancer is a major cause of cancer deaths in Western countries and is associated with diets high in red meat. Heme, the iron-porphyrin pigment of red meat, induces cytotoxicity of gut contents which injures surface cells leading to compensatory hyperproliferation of crypt cells. This hyperproliferation results in epithelial hyperplasia which increases the risk of colon cancer. In humans, a high red-meat diet increases *Bacteroides* spp in feces. Therefore, we simultaneously investigated the effects of dietary heme on colonic microbiota and on the host mucosa of mice. Whole genome microarrays showed that heme injured the colonic surface epithelium and induced hyperproliferation by changing the surface to crypt signaling. Using 16S rRNA phylogenetic microarrays, we investigated whether bacteria play a role in this changed signaling. Heme increased Bacteroidetes and decreased Firmicutes in colonic contents. This shift was most likely caused by a selective susceptibility of Gram-positive bacteria to heme cytotoxic fecal water, which is not observed for Gram-negative bacteria, allowing expansion of the Gram-negative community. The increased amount of Gram-negative bacteria most probably increased LPS exposure to colonocytes, however, there is no appreciable immune response detected in the heme-fed mice. There was no functional change in the sensing of the bacteria by the mucosa, as changes in inflammation pathways and Toll- like receptor signaling were not detected. This unaltered host-microbe cross-talk indicates that the changes in microbiota did not play a causal role in the observed hyperproliferation and hyperplasia.

## Introduction

Colon cancer is a leading cause of cancer deaths in Western countries [1]. The risk to develop colon cancer is associated with diets high in red meat [2], but not with diets high in white meat, such as poultry and fish [3], [4]. Heme, the iron porphyrin pigment, is present at much higher levels in red- compared to white meat. Several epidemiological studies show that increased heme intake is related to increased colon cancer risk [5], [6]. Zhang et al [7], however, did not observe this association, but they did not account for protective factors such as the heme antagonist chlorophyll present in green vegetables [6].

Our previous studies show that when rodents consume heme, their colonic contents become more cytotoxic [8], [9]. This increased cytotoxicity injures colon surface epithelial cells and leads to initiation of hyperproliferation from stem cells in the crypts to compensate for the injured surface cells. Recently, we showed that dietary heme changed the surface to crypt signaling by downregulating feedback inhibitors of proliferation such as Wnt inhibitory factor 1 (Wif1), Interleukin-15 (IL-15), Indian Hedgehog (Ihh) and Bone morphogenetic protein 2 (Bmp2) in the surface epithelium [9].The resulting compensatory hyperproliferation and hyperplasia increases the risk of mutations in oncogenes and tumor suppressor genes and thereby increases the risk to develop colon cancer.

Dietary heme is poorly absorbed in the small intestine, and approximately 90% of dietary heme proceeds to the colon where it can be used by colonic bacteria as a growth factor [10]. The relationship between intestinal microbiota and development of colon cancer has long been suspected [11], especially since the colon is the most densely bacteria-populated intestinal region. Observational studies indicate that individuals with a high colon cancer risk have distinct fecal microbiota compared to healthy individuals (see [12] for review). Besides beneficial components such as short chain fatty acids, microbiota can also produce deleterious components, such as secondary bile acids [13], hydrogen sulfide from sulfate and sulfur-amino acids [14], and N-nitroso-compounds (NOC) from nitrite [15], which all might play a role in development of colon cancer [16], [17].

Recently, we showed by classical culturing methods that a heme diet increases the colonic enterobacteria in rats, but decreases lactobacilli [18]. Subsequently, the current manuscript explores how heme affects the overall composition of the gut microbiota by using a phylogenetic microarray specific for mouse gut-phylotypes. Whole genome microarrays were used to validate the simultaneous effects of heme on mouse colonic mucosa. Subsequently, we investigated whether the changes in microbiota can be related to the heme-induced epithelial hyperproliferation and hyperplasia.

## Materials and Methods

### Ethics statement

The institutional and national guidelines for the care and use of animals were followed and the experiment was approved by the Local Committee for Care and Use of Laboratory Animals at Wageningen University.

### Animals and diets

Eight-week-old male C57BL6/J mice (Harlan, Horst, the Netherlands) similar in weight (25.2 g±0.7 and 24.8 g±0.6 for heme and control respectively (mean ± SEM)) were individually housed in a room with controlled temperature (20–24°C), relative humidity (55%±15%) and a 12 h light-dark cycle. Mice were fed diets and demineralized water ad libitum. To study the effects of heme on the colonic epithelium, mice (n = 8/group) received either a Westernized control diet (40% fat (mainly palm oil) low calcium (30 µmol/g)) or this diet supplemented with 0.5 µmol/g heme (Sigma-Aldrich Chemie, St. Louis, USA) for 14 days, as previously described [19]. Feces were quantitatively collected during days 11–14, frozen at −20°C and freeze-dried. After 14 days of diet intervention the colon was excised, mesenteric fat was removed and the colon was opened longitudinally. Luminal colonic contents were collected and stored at −80°C for microbiome analysis. The colon was washed in PBS and cut into three parts. The middle 1.5 cm tissue was formalin-fixed and paraffin embedded for histology. The remaining proximal and distal parts were scraped. Scrapings were pooled per mouse, snap-frozen in liquid nitrogen and stored at −80°C until further analysis. The present study was an exact repetition of our recently described study [9], except for the microbiome analysis.

### Fecal analyses

Fecal water was prepared by reconstituting freeze-dried feces with double distilled water to obtain a physiological osmolarity of 300 mOsm/l, as described previously [8]. The cytotoxicity of fecal water was quantified by potassium release from human erythrocytes after incubation with fecal water [8]. The relevance of this bioassay was validated with human colon carcinoma-derived Caco-2 cells [20]. Fecal host DNA was determined as marker for epithelial exfoliation and quantification was based on real-time polymerase chain reaction as previously described [21]. To determine lipid peroxidation products in the colonic lumen, thiobarbituric acid reactive substances (TBARS) in fecal water were quantified (see supporting information).

### Histology

Immunohistochemical and immunofluorescent stainings were performed on paraffin embedded colon sections (details in supporting information). Ki67 immunohistochemistry was performed to stain proliferating cells, as described previously [9]. Colonocytes from 15 well-oriented crypts (longitudinal section) were counted for each animal.

### RNA isolation and microarrays

RNA was isolated from colon scrapings of n = 4 mice per group. The Ambion WT Expression kit (Life Technologies, P/N 4411974) in conjunction with the Affymetrix GeneChip WT Terminal Labeling kit (Affymetrix, Santa Clara, CA; P/N 900671) was used for the preparation of labeled cDNA from 100 ng of total RNA without rRNA reduction. Labeled samples were hybridized on Affymetrix GeneChip Mouse Gene 1.1 ST arrays, provided in plate format. Hybridization, washing and scanning of the array plates was performed on an Affymetrix GeneTitan Instrument, according to the manufacturer's recommendations. Array data was analyzed using an in-house, on-line system [22]. Briefly, normalized expression estimates were obtained from the raw intensity values applying the robust multi-array analysis (RMA) preprocessing algorithm [23], [24], available in the Bioconductor library AffyPLM with default settings. Probe sets were redefined according to Dai et al. [25]. In this study probes were reorganized based on the Entrez Gene database, build 37, version 2 (remapped CDF v15.1). Probe sets that satisfied the criterion of a False Discovery Rate (FDR)<1% (q-value<0.01) were considered significantly regulated and used for bioinformatics analysis by Ingenuity (Ingenuity® Systems, www.ingenuity.com). The number of mice included in the array analysis were n = 4 per group. Genes with signal intensities below 20 in all mice from all treatments were considered absent and excluded from further analysis. Array data were submitted to the Gene Expression Omnibus, accession number GSE40672.

The microarray procedure applied on LCM-isolated surface and crypt cells was previously described [9]. These array data were earlier submitted to the Gene Expression Omnibus, accession number GSE27849.

### Bacterial DNA extraction and MITChip procedure

DNA was extracted from 0.1 g of fresh colonic sample using the repeated bead beating method [26]. Microarray analyses were conducted by using the Mouse Intestinal Tract Chip (MITChip) produced by Agilent technologies (Agilent Technologies, Palo Alto, CA, USA) in analogy to the (previously developed) human HITChip [27]. Details about DNA extraction and MITChip are in the supporting information. A customized classification was introduced based on three levels of taxonomic resolution; Level 0 (phylum), Level 1 (class or *Clostridium* cluster) and Level 2 (including sequences with ≥90% sequence similarity, reflecting a genus-like level). The pre-processing was performed using in-house MySQL and R scripts. Ward's minimum variance method was used to generate hierarchical clustering of the total microbiota probe profiles by calculating a distance matrix between the samples based on the Pearson's distance [28].

### Quantitative PCR

qPCR included quantification of total bacteria using 16 S rRNA-specific primers and gene specific qPCR, targeting functional genes representing bacterial groups with the following capacity: sulfate or nitrate reduction. For detailed method see supporting information. Primer sequences can be found in Table S1.

### Bacterial incubations with fecal water

Gram-negative *Escherichia coli* (ABLE K) was cultivated in Luria-Bertani (LB) broth and Gram-positive *Lactobacillus plantarum* WCFS1 [29] was grown in MRS liquid medium (Becton Dickinson, Breda, the Netherlands). Bacteria from overnight cultures were pelleted by centrifugation for 15 min at 4800 rpm and washed 2 times with PBS. Cell-pellets were resuspended to an OD_600_ of 1, and 5 µl of the resulting suspension was mixed in 200 µl with 10 µl PBS or deoxycholate (final concentrations of 0.7, 1.3 and 2.7 mM) as controls, or with pooled control or heme fecal water from 4 biologically independent, but identical experiments of at least 6 mice per treatment . Each mixture was made in triplicate, and incubated for 2 h at 37°C. Subsequently, bacteria present in the suspensions were stained by adding live/death medium (BacLight Bacterial Viability and Counting Kit,Molecular Probes, Leiden, the Netherlands to a final volume of 200 µl) and analyzed by flow cytometry (FACS) analysis.

### Western blot

Homogenates of colon tissue were made by adding 1 ml of PBS containing protease inhibitors (CompleteMini, Roche Diagnostics, Mannheim, Germany) to part of a mucosal scraping of one animal. Tissue was homogenized using a turrax. Homogenates were centrifuged for 10 min at 4°C at 20.000×*g* and supernatant was collected. For Western blot analysis homogenates were pooled for n = 8 mice per group. Samples were applied to SDS-PAGE (17.5% gel) and transferred to polyvinylidene fluoride membranes. Ten µg total protein was loaded for detection of Slpi and β-actin. Membranes were blocked with Tris-buffered saline with 0.1% Tween20 and 5% milk powder (ELK). The blot was cut in two at around 26 kDa. The lower half containing proteins with sizes lower than 26 kDa was probed overnight at 4°C with antibodies against mouse Slpi (R&D systems, Minneapolis, USA 1∶500). The blot was incubated with peroxidase-conjugated donkey anti-goat antibody (Jackson ImmunoResearch, USA, 1∶5000) at room temperature for 1 h. The upper half of the blot containing the proteins with sizes higher than 26 kDa was probed overnight at 4°C with antibodies against β-actin (Sigma-Aldrich, St. Louis, USA 1∶3000). The blot was incubated with peroxidase-conjugated goat anti-rabbit antibody (Jackson ImmunoResearch, USA, 1∶5000) at room temperature for 1 h. The signals were detected with the enhanced chemiluminescence detection system (Amersham ECL, GE Healthcare, Little Chalfont, UK).

### Statistical analysis

All values are presented as mean ± SEM. Physiological responses,were compared using an unpaired, two-tailed t-test. Statistical analysis was carried out in SPSS Statistics 17.0 (SPSS Inc., Chicago, IL). A p-value<0.05 was considered as statistically significant. Statistical considerations for microarray analysis are described above. Microbiota analysis was carried out on different taxonomical levels: level 0 corresponding to phylum-like level, level 1 corresponding to class-like level and level 2 corresponding to genus-like level. Mann whitney analysis between heme and control groups, followed by correction for multiple testing by Benjamini Hochberg correction [30] was carried out. A q-value<0.05 was considered statistically significant.

## Results

### Effects of dietary heme on colon physiology

**Figure 1:**
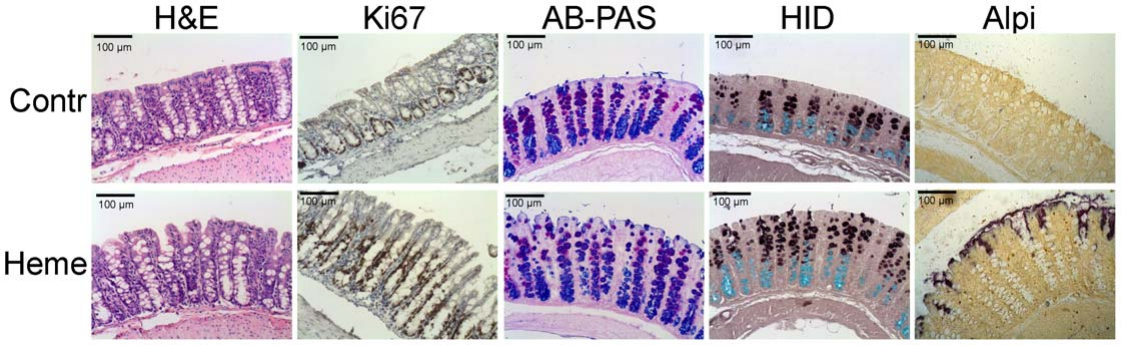
Histological staining of mouse colonic mucosa after 14 days on control or heme diet. Histological staining was performed for H&E, AB-PAS, HID and Alpi. Ki67-immunohistochemistry was performed using a Ki67-specific antibody.

Addition of heme to the diet for 2 weeks increased cytotoxicity of fecal water (Table 1). Besides cytotoxic stress, heme-fed mice suffered from Reactive Oxygen Species (ROS) stress in their colon, as shown by the increase of thiobarbituric acid reactive substances (TBARS) (Table 1). Dietary heme increased epithelial cell proliferation as shown by the Ki67-staining (Figure 1 and Table 1). Mucosal gene expression changes induced by heme were similar to those described into detail previously [9]. There was a high correlation between the current and the previous experiment (Pearson r = 0.896 with p<0.000 including only the significant changed genes (n = 3640, q<0.01). In the current as well as in the previous study, heme upregulated stress response genes such as Hmox1 (Table 1), Catalase and Glutathione related genes (not shown). Signaling from the injured surface epithelium to the proliferative crypt to initiate compensatory hyperproliferation occurs via downregulation of feedback inhibitors such as Wif1, IL-15, Ihh and Bmp2 [9]. Table 1 shows that, also in the current study, Wif1, IL-15 and Ihh were significantly downregulated by heme whereas Bmp2 was borderline significantly downregulated (q = 0.011). Cell cycle genes were induced (e.g. Ki67, Cyclins) and apoptosis inhibitors were upregulated (Birc5, Ier3) (Table 1).

**Table 1:** Differential effects of dietary heme on fecal and mucosal parameters.

	Control	Heme
**Feces**		
Cytotoxicity of fecal water (% lysis)	2±2	106±4 *
Fecal host DNA (µg/d)	1.31±0.25	0.48±0.09 *
Fecal TBARS (µmol/L malondialdehyde equivalents)	20.9±1.7	55.8±3.7 *
**Mucosal histology**		
Total number of cells per crypt	48.0±3.1	75.4±4.3 *
Number of Ki67 positive cells per crypt	19.7±2.6	44.3±3.5 *
Labeling index (% Ki67 positive cells per crypt)	40.5±2.2	58.8±1.6 *
**Mucosal gene expression**		
Heme oxygenase 1 (Hmox1)	86±5	923±271*
Wnt Inhibitory Factor 1 (Wif1)	63±3	48±2*
Interleukin-15 (Il-15)	399±7	169±4*
Indian Hedgehog (Ihh)	655±35	487±35*
Bone morphogenetic protein (Bmp2)	1075±160	615±82
Ki67 (Mki67)	1318±63	2193±57*
Cyclin E1 (Ccne1)	145±19	265±16*
Cyclin A2 (Ccna2)	1123±116	2262±79*
Cyclin B2 (Ccnb2)	1414±142	2387±85*
Immediate Early response 3 (Ier3)	127±12	185±22
Survivin, baculoviral IAP repeat-containing 5 (Birc 5)	117±9	230±10*
Receptor-interacting protein (Ripk3)	330±18	556±46*

Data are represented as mean ± SEM, n = 8 for feces and mucosal histology and n = 4 for gene expression.

* Significantly different from respective controls at q<0.01 for gene expression and p<0.05 for other parameters.

In line with the earlier described inhibition of apoptosis [21], we found that heme upregulated the necrosis inducer receptor interacting protein kinase-3 (Ripk3) by 1.7-fold, indicating that epithelial cells die predominantly by necrosis [31]. This would result in a diminished, apoptosis-dependent, cell shedding [32] and thus decreased levels of host DNA in feces. Therefore we measured host DNA levels of the feces of control and heme fed mice. The amount of host DNA in the feces of heme-fed mice was significantly lower compared to controls (Table 1).

### Effects of dietary heme on microbial composition

**Figure 2:**
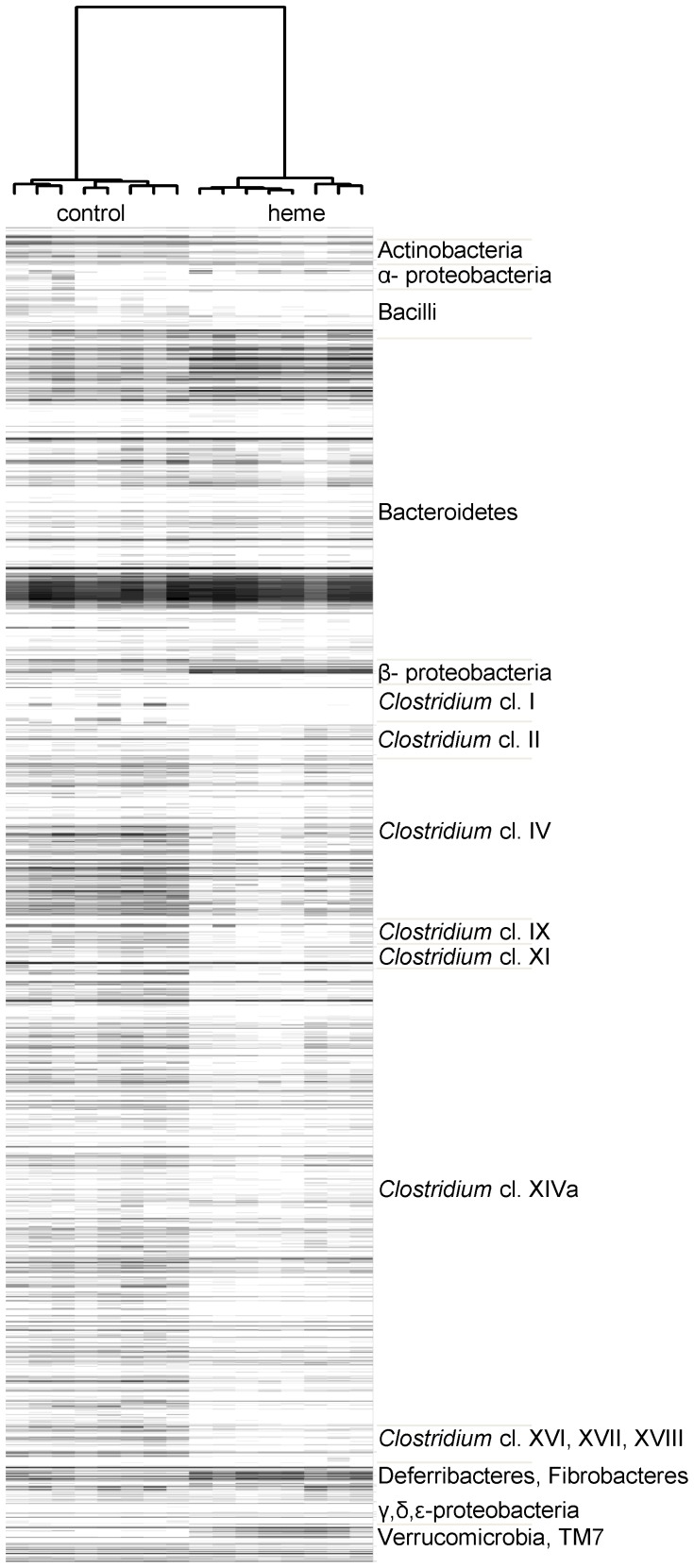
Phylogenetic fingerprints of the colonic microbiota of the control group and heme group. Gel-view figure of the intensity of 3,580 probes covered by MITChip and assigned to the phylogenetic class-like groups (level 1) depicted on the right side. Ward's minimum variance method was used to generate hierarchical clustering of the total microbiota probe profiles, whereas the distance matrix between the samples was based on the Pearson's product moment correlation.

Until now, the effect of heme or red meat on colonic bacteria was studied only by classical culturing methods [18]. Therefore, we now investigate whether the overall composition of colonic microbiota of heme-fed mice differed from that of control mice by using 16S rRNA phylogenetic microarrays, targeting murine phylotypes (MITChip). The MITChip has previously been used to study the microbiota from mouse intestinal samples [33]. In our study, MITChip analysis indicated that samples of heme-fed mice clustered separately from control mice (Figure 2), because of major changes in bacterial phyla (Figure S1) and classes (Table 2). Upon heme consumption, 12 bacterial classes were significantly changed. Amongst them, the Gram-negative Bacteroidetes, Beta- and Epsilon-Proteobacteria and Verrucomicrobia were increased, while the Gram-positives such as the Actinobacteria and Firmicutes, including Bacilli, and *Clostridium* clusters IV and XIVa, were decreased in heme-fed mice. A further analysis of the bacterial composition was carried out at genus-level (designated level-2 in the chip data; Table S2). Genera such as *Bacteroides*, *Alistipes*, *Prevotella*, *Helicobacter*, *Sphingomonas*, *Akkermansia* were significantly increased upon heme-feeding. Based on MITChip data, Gram-negative bacteria (i.e. Bacteroidetes, Deferribacteres, Fibrobacteres, Fusobacteria, Proteobacteria and Verrucomicrobia) accounted for 40.7±3.5% of the total bacteria in the controls, while the heme diet promoted an increase (p<0.001) of relative abundance of Gram-negative bacteria to 66.6±3.2% of the total bacteria detected. The total bacterial density was similar between the heme and control group (Table 3), indicating that a heme-diet induced drastic changes in the ratio of Gram-negative to Gram-positive bacteria from 0.7 to 2.2 (Table 3) without influencing the overall community density. Analogously, the ratio between the most dominant Gram-negative and Gram-positive phyla, Bacteroidetes and Firmicutes, was also strongly increased (Table 3).

**Table 2:** Relative contribution of bacterial classes- *Clostridium* clusters (level 1) detected by MITChip in control and heme-fed mice.

Phylum	Class	Control	Heme
**Actinobacteria**	Actinobacteria	2.46±0.32	1.26±0.04**
**Bacteroidetes**	Bacteroidetes	33.48±3.71	53.11±4.77*
**Firmicutes**	Bacilli	1.15±0.13	0.80±0.06*
	*Clostridium* cluster I	0.97±0.19	0.67±0.05
	*Clostridium* cluster II	1.55±0.11	1.17±0.18
	*Clostridium* cluster IV	10.83±0.79	5.01±0.67**
	*Clostridium* cluster IX	0.28±0.04	0.03±0.00**
	*Clostridium* cluster XI	3.01±0.38	2.20±0.47
	*Clostridium* cluster XIVa	25.76±1.71	12.33±1.87**
	*Clostridium* cluster XVI	4.24±0.80	1.98±0.09**
	*Clostridium* cluster XVII	1.60±0.20	1.63±0.14
	*Clostridium* cluster XVIII	3.66±0.51	3.23±0.28
	Mollicutes	2.93±0.41	2.62±0.24
**Deferribacteres**	Deferribacteres	1.40±0.35	1.38±0.38
**Fibrobacteres**	Fibrobacteres	0.19±0.03	0.15±0.02
**Fusobacteria**	Fusobacteria	0.80±0.07	0.70±0.12
**Proteobacteria**	Proteobacteria (alpha)	0.74±0.05	1.26±0.23
	Proteobacteria (beta)	0.57±0.08	2.05±0.22**
	Proteobacteria (delta)	0.21±0.06	0.10±0.04
	Proteobacteria (epsilon)	0.26±0.02	0.56±0.06**
	Proteobacteria (gamma)	2.83±0.20	4.99±1.38
**TM7**	TM7	0.88±0.07	0.48±0.09*
**Verrucomicrobia**	Verrucomicrobia	0.18±0.07	2.28±0.63*

Data are represented as mean ± SEM (n = 8),

* q<0.05,

** q<0.01.

significance indicated between the control group and the heme group tested by Mann Whitney corrected for multiple testing by Benjamini-Hochberg procedure.

**Table 3:** Quantification of total bacteria and specific functional genes in colonic samples from control and heme-fed mice.

	Control	Heme
**Total bacteria (16S rRNA)**	11.20±0.08	11.30±0.17
**Ratio Gram-negative to Gram-positive bacteria**	0.72±0.09	2.16±0.27 **
**Ratio Bacteroidetes to Firmicutes**	0.64±0.09	1.88±0.30*
**Sulfate reducers (** ***DsrA*** **)**	7.92±0.15	8.29±0.15
**Nitrate reducers (** ***NarG*** **)**	9.45±0.16	10.50±0.13*

Data are represented as mean ± SEM, (n = 8).

* indicates significant change between control and heme (p<0.05),

** indicates significant change between control and heme (p<0.001).

For ratio calculation, the in Table 2 quantified Actinobacteria, Firmicutes and TM7 were included as Gram-positives and Bacteroidetes, Deferribacterres, Fibrobacteres, Fusobacteria, Proteobacteria and Verrucomicrobia were included as Gram-negatives.

Results are expressed in log10 of gene copy-nr/g fecal dry weight. Genes used for quantification are mentioned between brackets.

Next we investigated whether heme can act as a selective growth factor involved in the modulation of the ratio of Gram-negative to Gram-positive bacteria. Notably, several members of the Gram-negative Bacteroidetes can use heme as a growth factor [34]. However, such a generic growth stimulating effect of heme was unlikely, since several Gram-positive bacteria able to use heme [35], such as *Corynebacterium diphteriae et rel.* (fold-change = 0.8), and *Streptococcus* (fold-change = 0.13), were decreased. Alternatively, various bacteria can use heme as electron carrier in cytochrome- catalyzed anaerobic respiration, like sulfate and nitrate reduction in anaerobic bacteria. Therefore, putative changes in sulfate and nitrate reduction capacity in the microbiota where assessed by quantification of the corresponding functional gene copy-numbers within the total microbiota community. This was done by qPCR for dissimilatory sulfate reductase (*dsrA*) and the membrane-bound nitrate reductase (*narG*) encoding genes. There was no significant difference in abundance of the *dsrA* gene between the control and heme-fed group (Table 3), suggesting an unaltered sulfate-reduction capacity of the microbiota. In contrast, the abundance of the *narG* gene was significantly higher in the microbiota of the heme-fed mice, indicating an enhanced microbial nitrate reduction capacity. However, nitrate reduction occurs in various Gram-negative as well as -positive bacteria, implying that increased nitrate reduction capacity in the microbiota encountered on the heme diet cannot be correlated directly with the increased number of Gram-negative bacteria. Taken together, these results make it unlikely that heme selectively stimulates the growth of Gram-negative bacteria and thereby changes the ratio of Gram-negative to Gram-positive bacteria.

**Figure 3:**
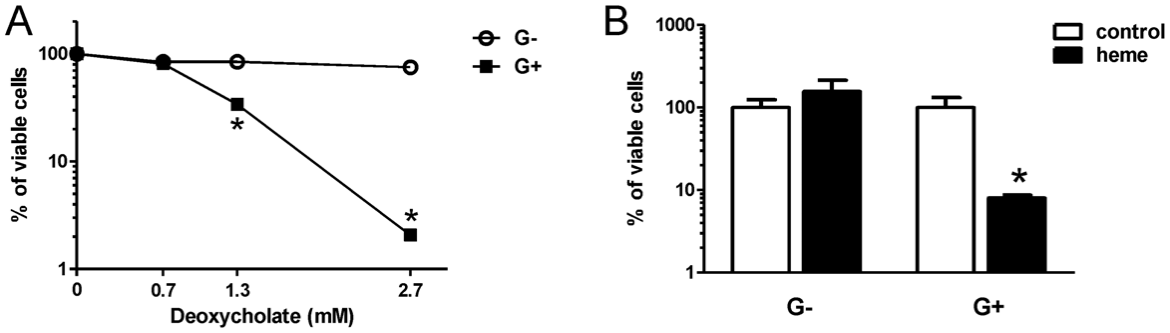
Differential effect of deoxycholate (A) and of control and heme fecal water (B) on viability of Gram-negative (G−) *E.coli* and Gram-positive (G+) *L. plantarum*. Fecal water pools from four separate, but identical, animal experiments were used in triplicate. Viability is measured relative to PBS (A) and control fecal water (B). Data are presented as mean ± SEM, (n = 4) *p<0.05.

Another possibility is that the ratio of Gram-negative to Gram-positive bacteria is directly affected by the toxic heme, which may act as a lytic surfactant to which Gram-positive bacteria are more susceptible, thereby selectively decreasing the Gram-positive bacterial populations, and allowing expansion of the Gram-negative community [36]. To evaluate whether the cytotoxic heme had a differential impact on the viability of Gram-negative and Gram-positive bacteria, the exemplary Gram-negative *Escherichia coli* (ABLE K), and Gram-positive *Lactobacillus plantarum* WCFS1 were incubated with fecal water of heme or control mice, or with deoxycholate as a positive control. There was a clear concentration-dependent decrease of viability of Gram-positive *L. plantarum* cells when these were incubated with deoxycholate (Figure 3A). However, the percentage of viable Gram-negative *E. coli* cells was barely affected by deoxycholate treatment. Similarly, *L. plantarum* was more sensitive to fecal water from heme-fed mice as compared to the control, whereas the viability of *E. coli* was equally affected by heme and control fecal water (Figure 3B). These differential sensitivity effects were consistently found for fecal water pools from four biologically independent, but similar diet-interventions in mice, suggesting that heme fecal water has a reproducible antimicrobial effect on our Gram-positive model-bacterium. Assuming that our model bacteria represent the generic difference between the cell membranes of Gram-positive and Gram-negative bacteria, these in-vitro results may explain the observed modulation of the ratio between Gram-negative and Gram-positive bacteria in the colon microbiota of heme- and control-fed mice.

### Heme did not change microbe-sensing pathways in colonic mucosa

Because of these drastic changes in colon microbiota we queried the mucosa for heme-induced differences in inflammation markers, mucin dynamics and Toll-like receptor (Tlr) signaling pathways. The surface epithelium of the heme fed mice was ruffled, because of luminal necrosis [21], but there were no signs of inflammation, as there was no infiltration of neutrophils or macrophages observed in the lamina propria (Figure 1). Moreover, the expression of inflammation markers for macrophages (CD14, CD68, CD11b, and F4/80,) and for neutrophils (Mpo, lactoferrin, neutrophil elastase, and Emr4) was not changed by dietary heme (Table 4). Next we studied mucins as they protect the surface epithelium against bacteria and toxic luminal factors and are increased by inflammation [37]. There were no clear differences in presence and distribution of neutral and acidic mucins between the heme-fed animals and the control animals as shown by AlcianBlue/PAS staining (Figure 1). The same holds for the sulfated and carboxylated mucins shown by HID staining (Figure 1). The gene expression of secreted Muc2 was not changed (Table 4), whereas that of the cell-associated Muc1, 3 and 13 was significantly downregulated. Thus, the heme diet did not increase mucin turnover that is typical of inflammation.

**Table 4:** Mucosal gene expression on control or heme diet.

Gene name	Symbol	Signal intensity control	Signal intensity heme
**Macrophage markers**			
CD14 antigen	Cd14	270±30	267±37
CD68 antigen	Cd68	205±18	176±13
integrin alpha M	Itgam/Cd11b	84±10	93±7
EGF-like module containing, mucin-like, hormone receptor-like sequence 1	Emr1/F4/80	127±15	109±12
**Neutrophil markers**			
myeloperoxidase	Mpo	23±2	23±2
lactotransferrin	Ltf	21±1	22±2
elastase, neutrophil expressed	Elane	25±2	23±2
EGF-like module containing, mucin-like, hormone receptor-like sequence 4	Emr4	15±2	13±0
**Mucins**			
Mucin 1	Muc1	190±32	56±5*
Mucin 2	Muc2	11040±182	10301±199
Mucin 3	Muc3	8429±108	7017±112*
Mucin 4	Muc4	1100±68	1191±104
Mucin 13	Muc13	6845±68	5999±26*
**Anti-microbial response**			
Alkaline phosphatase, intestinal	Alpi	746±114	1691±59*
Secretory leukocyte peptidase inhibitor	Slpi	110±2	1612±218*
Regenerating islet-derived 3 beta	Reg3β	1770±852	1150±626
Regenerating islet-derived 3 gamma	Reg3γ	1036±480	844±334
Lysozyme	Lyzs	1523±62	1580±88
**Toll-like receptor signaling**			
Toll-like receptor 1	Tlr1	1713±47	1375±49*
Toll-like receptor 2	Tlr2	444±48	219±7*
Toll-like receptor 4	Tlr4	309±14	242±13*
Toll-like receptor 6	Tlr6	31±1	35±1
Toll-like receptor 9	Tlr9	58±2	61±3
Inhibitor of kappaB kinase gamma	Ikbkγ	315±9	588±39*
Tnf receptor-associated factor 6	Traf6	493±16	549±24
Toll-interleukin 1 receptor (TIR) domain-containing adaptor protein	Tirap	447±30	506±13
Myeloid differentiation primary response gene 88	Myd88	842±37	904±32
Tumor necrosis factor	Tnfα	54±3	50±1
Interleukin 1 beta	Il-1β	40±3	36±2
Interleukin 6	Il-6	n.e.	n.e.
Interleukin 12a	Il-12a	23±1	25±1
Interleukin 12b	Il-12b	30±1	28±2

* indicates significant fold change (q<0.01), genes with signal intensities below.

20 are considered not expressed (n.e.).

Colonocytes can also directly respond to bacterial cell components via Tlr-signaling pathways. The strong increase in Gram-negative bacteria in colon microbiota of heme-fed mice suggests an increased exposure of the mucosa to lipopolysaccharide (LPS). The expression of Tlr4, which recognizes LPS, was downregulated with a fold-change of −1.3 (Table 4). Tlr2 can form heterodimers with Tlr1 or with Tlr6 to detect different ligands (reviewed in [38] ). Tlr2/1 recognizes triacyl lipoproteins found mainly in Gram-negative bacteria, while Tlr2/6 recognizes diacyl groups on lipoteichoic acid and lipoproteins of Gram-positive bacteria. The heme intervention downregulated the expression of Tlr1 and Tlr2 (fold-change resp. −1.2 and −2.0, Table 4), but Tlr6 remained unchanged. Downstream targets of TLRs, such as MyD88, Traf6 and Tirap, were not changed, except for the NF-κB activator Nemo (Ikbkγ), which was induced by heme (Table 4).

Although the heme diet led to, (i) increased exposure to Gram-negative bacteria, (ii) altered expression of Tlr1, 2 and 4, as well as (iii) Nemo, these changes did not elicit significant modulation of the transcription of downstream genes associated with inflammatory cytokine production, such as TNFα, IL-1β, IL-6 and IL-12. This indicated that the heme diet did not induce functional changes in the Tlr-signaling pathways (Table 4). Moreover, Serum Amyloid A3 (Saa3), a gene encoding a protein involved in acute phase response, is 8-fold downregulated in the mucosa of the heme-fed animals (data not shown), supporting the lack of innate immune responses in heme-fed mice. Finally, plasma IgM against core endotoxin was measured in this study and its concentration was similar in heme-fed and control mice (not shown), indicating that also a systemic marker for immune activation was unaffected by heme. Taken together these results show an unaltered innate immune system in the heme-fed mice as compared to the controls.

**Figure 4:**
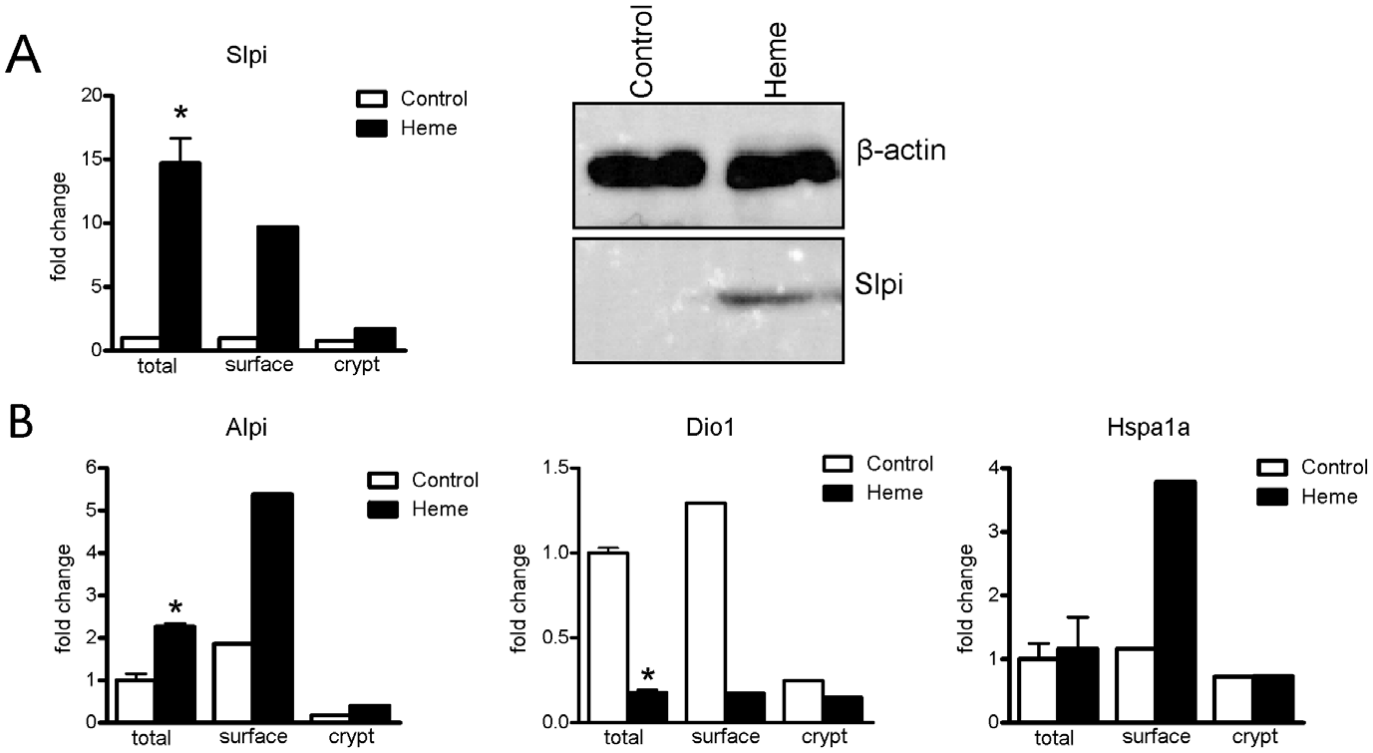
Gene expression and protein levels of Slpi and gene expression levels of Alpi, Dio1 and Hspa1a. Western blot (A) shows the presence of Slpi protein colon scraping homogenate pools (n = 8 for both groups) of heme-fed mice. Slpi is not detectable in controls. β-actin serves as a loading control. (B) Gene expression levels (A and B) in total scrapings are based on microarray analysis on individual mice (n = 4 per group). Relative gene expression changes (fold-changes) on heme were calculated by setting RNA levels of control mice to 1 (mean ± SEM, *q<0.01). Surface- and crypt-specific microarray gene expression profiles are from pooled tissue samples obtained by LCM (n = 4 controls, n = 3 heme-fed mice). In this LCM experiment also total scrapings were again collected and analyzed by microarray. Surface and crypt expression values are normalized for the expression in total scrapings.

The absence of a heme-induced innate immune response is in good agreement with the observation that the heme diet did not increase macrophage and neutrophil recruitment in the mucosa (see above), and is also in line with the unchanged expression of well-known anti-microbial peptides (defensins (not shown), lysozyme and regenerating islet-derived 3 in the current study (Table 4)). Interestingly, the heme diet led to a drastic increase of expression of Secretory Leukocyte Protease Inhibitor (Slpi), which was especially prominent in the surface epithelium (Figure 4A). Western blot analysis confirmed the elevated Slpi levels in colon homogenates obtained from heme-fed mice as compared to control-fed mice (Figure 4A). Finally, we evaluated whether the mucosal surface did not sense the increased number of Gram-negative bacteria due to LPS inactivation by Alkaline phosphatase (Alpi), which can dephosphorylate LPS and thereby preventing its induction of Tlr4-signaling [39]. Indeed mucosal Alpi is 2.5-fold upregulated in the epithelium of heme-fed mice, specifically in the surface epithelium (Figure 4B). This transcriptional effect is validated by histochemical analysis showing that heme increases the activity of this apical ecto-enzyme, especially in the surface epithelium (Figure 1). Alpi expression can be induced by thyroid hormone or cellular stress, reviewed in [40]. It is unlikely that thyroid hormone activation occurred in this study, because the heme diet led to decreased surface expression of the dioxygenase-enzyme (Dio1), which converts inactive thyroxin into locally active thyroid hormone. *In-vitro* studies show that induced Alpi expression through cellular stress co-occurs with Heat Shock Protein (Hsp)72 induction in enterocytes [41]. This is corroborated by our finding that heme also induced the expression of Hsp72 (Hspa1a), exclusively in the surface epithelium (Figure 4B), indicating that the induction of Alpi and Hsp72 can be part of the heme-induced stress response in the surface epithelium, which we recently described in detail [9]. The oxidative stress-induced Alpi may inactivate LPS and thus protect the epithelium against luminal LPS exposure.

## Discussion

This study shows that dietary heme not only impacts the colonic mucosa by inducing epithelial surface injury and compensatory hyperproliferation and hyperplasia, but also drastically changed the microbiota composition in mouse colon. This change is characterized by an increased ratio of Gram-negative to Gram-positive bacteria on the heme diet, which was predominantly caused by increased abundance of the Gram-negative bacteria *Bacteroides*, *Prevotella* (Bacteroidetes), *Helicobacter* (Gamma-proteobacteria), *Akkermansia* (Verrucomicrobia) and *Mucispirillum* (Deferribacteres). Interestingly, an increase in *Bacteroides* spp on a high-red meat diet has been previously reported in humans, which coincided with reduced abundance of lactobacilli and clostridia [42]. Moreover, *Bacteroides* spp were also more abundant in the colon of humans eating meat compared to vegetarians [43]. Thus, the findings of this study are in agreement with the changes observed in humans, illustrating similar microbiota modulations in humans consuming a high red-meat diet, and the mouse experiment reported here.

We studied functional bacterial genes previously reported to be linked with colon cancer, such as sulfate- and nitrate reduction. Sulfate reducing bacteria produce sulfide which is associated with an increased risk to develop colon cancer [44], [45]. However, changes in sulfate reduction capacity were not observed in our study, neither by changed abundance of the major genus of colonic sulfate reducers (*Desulfovibrio*) in the MITChip, nor by qPCR targeting the conserved gene involved in sulfate reduction. Nitrate reducing bacteria are also associated with colon cancer, as nitrite is a precursor in the production of the putatively carcinogenic N-nitrosocompounds (NOC). Diets high in red meat increased apparent NOC, which are able to form alkylating DNA adducts in the colon [15]. If these adducts are not repaired, this increases the risk of colon cancer. Microbiota may play a role in NOC formation as nitrate reduction is a common activity of the GI tract microbiota [46]. The nitrate reducing capacity was significantly increased in microbiota of heme-fed mice as shown by the elevated copy numbers of the conserved gene involved. This indicates that dietary heme, besides the previously reported cytotoxic and hyperproliferative effect [9], may have an additional carcinogenic effect in the colon that is mediated via the microbiota.

Our results suggest that the cause for the increased ratio of Gram-negative to Gram-positive bacteria is most probably related directly to the presence of toxic heme in fecal water of heme-fed mice. The *in-vitro* experiments presented support a selective susceptibility of Gram-positive bacteria to heme fecal water, which is not observed for Gram-negative bacteria, and is in agreement with the general higher susceptibility of Gram-positives to surfactants. There are no indications that factors such as anti-microbial peptides produced by the host mucosa influence this ratio directly. We speculate that the heme lipophilic moiety can enter the membrane of the Gram-positive bacteria and exerts its toxic effect through typical surfactant activity leading to loss of membrane integrity and viability, which also occurs during treatment with other cytotoxic surfactants such as bile acids [36]. Consequently, the selective reduction of Gram-positive members of the microbiota by the heme-derived surfactant activity, allows the generally more resistant Gram-negative bacteria to thrive and expand their relative (and absolute) abundance in heme-fed mice. It was suggested that Gram-positive bacteria are particularly vulnerable to the luminal surfactant concentration as they are lacking the outer LPS-containing membrane [36]. Our observed heme effect is in line with classical culture studies showing that heme preferentially kills Gram-positive bacteria [47].

More Gram-negative bacteria in the colon microbiota of heme-fed mice would expose the colonic epithelium to increased amounts of LPS, which is known to elicit innate immune responses (reviewed in [48]). However, despite the increased exposure to Gram-negative bacterial communities, there was no appreciable innate immune response detected in the colon of heme-fed mice. Plasma IgM against core endotoxin levels remained unaffected by the heme diet, whereas its elevation is a marker for general activation of the immune system. The downregulation of Saa3, involved in acute phase response, in the mucosa of heme-fed mice suggests that the immune responses in the mucosa are repressed rather than activated by heme. This is in agreement with the finding that heme inhibits the inflammatory response in TNBS-treated rats [18].

The suppression of an immune response might be attributed to Slpi induction exclusively found in heme-fed mice. Slpi may act as an antimicrobial peptide as well as a modulator of the response to LPS stimulation [49], [50]. Furthermore, Slpi is also shown to counteract excessive inflammatory responses [51]. Therefore, Slpi could play a major role in maintaining mucosal homeostasis to protect the host against the drastic changes in Gram-negative bacteria and thus LPS exposure after heme consumption. Slpi can be induced upon tissue injury [51] and therefore might be triggered by heme-induced surface damage in this study. The antimicrobial effect of Slpi has been shown with the Gram-negative target organisms *Salmonella typhimurium* and *E. coli* [52]. However, this contrasts with our observation that heme drastically increased the abundance of Gram-negative bacteria. Furthermore, although Slpi has been reported to be secreted apically [52], we were unable to detect Slpi in fecal water (not shown), suggesting that the effect of Slpi is exerted basolaterally (immune suppression), which would be in agreement with the previously reported lack of detectable Slpi in the colonic mucus layer of mice [53].

It was suggested that Alpi acts as a microbiota controlled LPS-detoxifying mechanism functioning at the apical surface [54]. This was based on the finding that in zebrafish Alpi was absent in germ-free animals, and its expression was induced following exposure to bacteria [55]. However, this induction may be zebrafish-specific since Alpi activation was not observed in germ-free mice and rats during conventionalization (reviewed in [40]). In our study, Alpi induction co-occurs with increased expression of Hsp72, indicating that Alpi induction is most probably part of the heme-induced epithelial stress response [9].

Because our experimental diet mimics the nutrient composition of a Western human diet our results may have implications for the human situation. In the present mice study we used the concentration of 0.5 µmol heme/g diet. In an earlier study we showed that 0.25 and 0.5 µmol heme/g diet have similar effects in a rat model [56].We have recently also tested 0.2 µmol heme/g on the same background diet in mice. This concentration increased cell proliferation to a similar extent as 0.5 µmol heme/g diet. Preliminary analysis of the array data of both concentrations shows similar patterns of up- and downregulated genes. As an average human diet consists of about 400 g dry weight per day, 0.2 µmol heme/g diet corresponds to 80 µmol heme per day. Because beef contains 0.5 µmol heme/g wet weight [57], this implies a realistic beef intake of about 160 g/day in humans.

In conclusion, our study shows that dietary heme changed the microbiota with a major increase in the ratio of Gram-negative to Gram-positive bacteria, which is most likely explained by the selective susceptibility of the Gram-positives to toxic heme fecal water. Furthermore, heme changed the colon mucosa of the host by inducing hyperproliferation and subsequently hyperplasia. It is especially remarkable that the increased amount of Gram-negative bacteria, which coincides with increased mucosal exposure to LPS, did not elicit a detectable change in Tlr4 mediated immune reaction in the host mucosa. This may be due to the strong upregulation of Slpi which is able to suppress excessive immune reactions. We did not detect any sign of heme-dependent inflammation or functional change in epithelial microbe sensing. This indicates that the change in microbiota does not cause the observed hyperproliferation and hyperplasia via inflammation pathways. Whether this may occur via alternative mechanisms in colon lumen, such as modulation of oxidative and cytotoxic stress, will be studied using broad-spectrum antibiotics.

## Supporting Information

Figure S1 **Relative contribution of phylum levels of the individual control (C) and heme-fed (H) mice, n = 8 mice per group.**
(TIF)

Table S1 Primers sequences used in this study.
(DOCX)

Table S2 Averaged relative contribution of significantly (q<0.05) changed genus-like groups (levels 2) detected by MITChip in control and heme-fed mice. Genera are ranked by their highest ratio heme/control.
(DOCX)

Materials and Methods S1 (DOC)
