# Supplementary figures and images for: Dietary Heme Alters Microbiota and Mucosa of Mouse Colon without Functional Changes in Host-Microbe Cross-Talk
Source: PLoS One. 2012 Dec 11;7(12):e49868. doi: 10.1371/journal.pone.0049868 (PMC3519815; doi:10.1371/journal.pone.0049868)

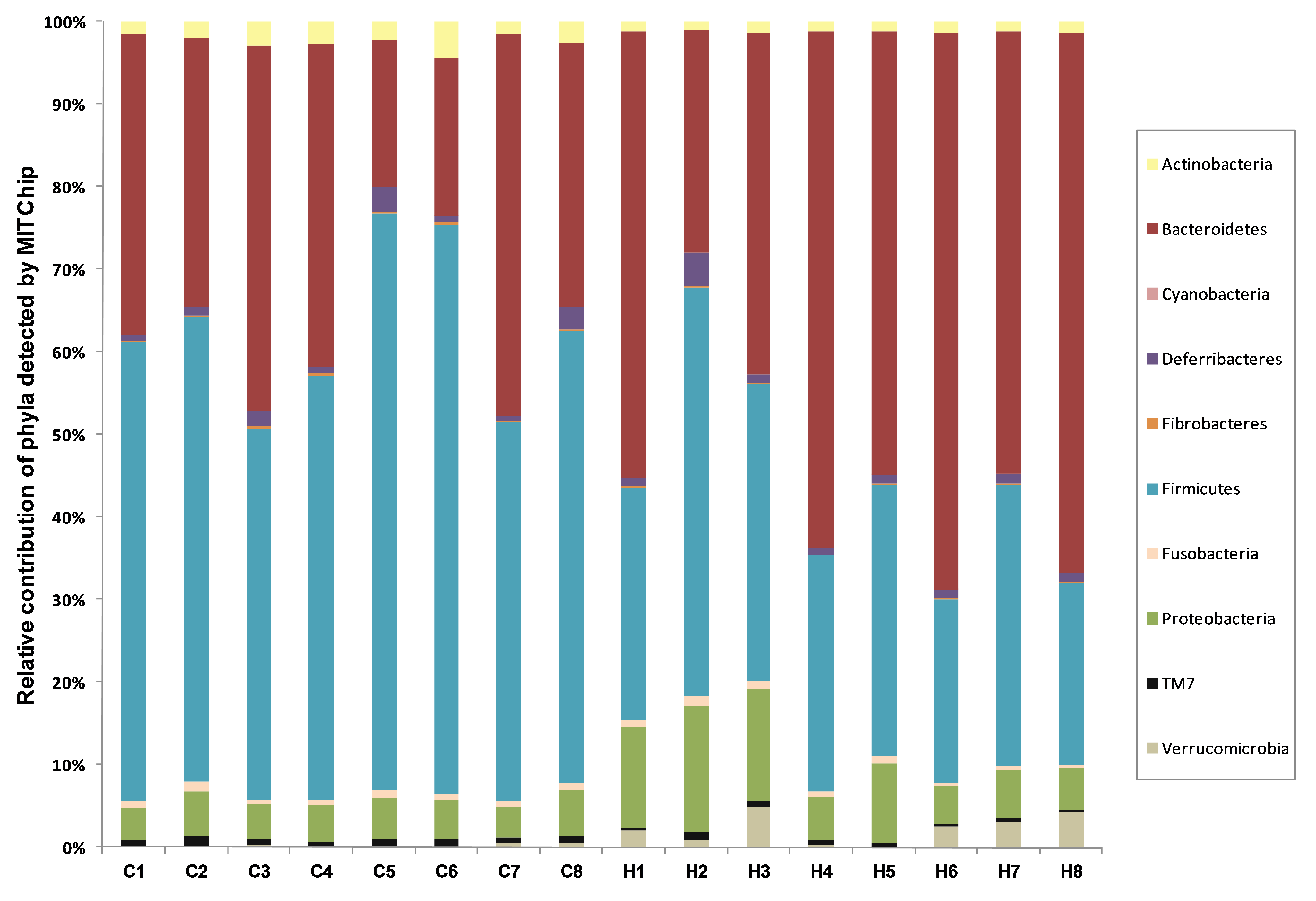

Supplement: Figure S1 — Relative contribution of phylum levels of the individual control (C) and heme-fed (H) mice, n = 8 mice per group. (TIF) [file pone.0049868.s001.tif]
