# Supplementary material for: Dietary Heme Alters Microbiota and Mucosa of Mouse Colon without Functional Changes in Host-Microbe Cross-Talk
Source: PLoS One. 2012 Dec 11;7(12):e49868. doi: 10.1371/journal.pone.0049868 (PMC3519815; doi:10.1371/journal.pone.0049868)
Supplement: Table S1 — Primers sequences used in this study. (DOCX) [file pone.0049868.s002.docx]

**Table S1.** Primers sequences used in this study**.**

| **Primer name** | **Primer sequence (5’-3’)** | **References** |
| --- | --- | --- |
| **16S Total bacteria (MITChip)** | | |
| T7 prom-Bact-27-F | TGAATTGTAATACGACTCACTATA GGGgtttgatcctggctcag | **[**[**2**](#_ENREF_2)**]** |
| Uni-1492-R | CGG CTA CCT TGT TAC GAC |  |
| **16S Total bacteria (qPCR)** | | |
| Bact-1369-F | CGGTGAATACGTTC | **[**[**4**](#_ENREF_4)**]** |
| Prok-1492-R | GGWTACCTTGTTAC |  |
| **Sulfate reducers** | | |
| RH1dsr-F | GCCGTTACTGTGACCAGCC | **[**[**5**](#_ENREF_5)**]** |
| RH3-dsr-R | GGTGGAGCCGTGCATGTT |  |
| **Nitroreducers** | | |
| narG-F | TCGCCSATYCCGGCSATGTC | **[**[**6**](#_ENREF_6)**]** |
| narG-R | GAGTTGTACCAGTCRGCSGAYTCSG |  |
