# Supplementary material for: Dietary Heme Alters Microbiota and Mucosa of Mouse Colon without Functional Changes in Host-Microbe Cross-Talk
Source: PLoS One. 2012 Dec 11;7(12):e49868. doi: 10.1371/journal.pone.0049868 (PMC3519815; doi:10.1371/journal.pone.0049868)
Supplement: Table S2 — Averaged relative contribution of significantly (q<0.05) changed genus-like groups (levels 2) detected by MITChip in control and heme-fed mice. Genera are ranked by their highest ratio heme/control. (DOCX) [file pone.0049868.s003.docx]

**Table S2.** Averaged relative contribution of significantly (q<0.05) changed genus-like groups (levels 2) detected by MITChip in control and heme-fed mice. Genera are ranked by their highest ratio heme/control.

| **Higher taxonomy level** | **Genus level** | **Relative abundance** | | | | | | **Ratio heme/ control** | **q-value** |
| --- | --- | --- | --- | --- | --- | --- | --- | --- | --- |
|  |  | **Control** | | | **Heme** | | |  |  |
| Verrucomicrobia | *Akkermansia* | 0.18 | ± | 0.07 | 2.28 | ± | 0.63 | 12.69 | 0.016 |
| Bacteroidetes | *B. distasonis et rel.* | 0.19 | ± | 0.03 | 2.29 | ± | 0.18 | 12.13 | 0.001 |
| Bacilli | *Enterococcus* | 0.01 | ± | 0.00 | 0.12 | ± | 0.05 | 9.14 | 0.030 |
| Bacteroidetes | *B. vulgatus et rel.* | 0.43 | ± | 0.10 | 3.56 | ± | 0.39 | 8.24 | 0.001 |
| Bacteroidetes | *Prevotella* | 0.40 | ± | 0.05 | 2.52 | ± | 0.19 | 6.37 | 0.001 |
| Proteobacteria | *Sphingomonas* | 0.23 | ± | 0.04 | 0.97 | ± | 0.24 | 4.22 | 0.011 |
| Bacteroidetes | *B. plebeius et rel.* | 0.18 | ± | 0.04 | 0.69 | ± | 0.08 | 3.90 | 0.002 |
| Proteobacteria | *Sutterella wadsorthia et rel.* | 0.57 | ± | 0.08 | 2.05 | ± | 0.22 | 3.60 | 0.001 |
| Bacteroidetes | *B. fragilis et rel.* | 1.47 | ± | 0.31 | 4.88 | ± | 0.60 | 3.33 | 0.002 |
| Bacteroidetes | *Alistipes* | 2.42 | ± | 0.37 | 5.86 | ± | 0.46 | 2.42 | 0.002 |
| Bacteroidetes | *Rikenella* | 0.43 | ± | 0.09 | 0.99 | ± | 0.07 | 2.30 | 0.003 |
| Proteobacteria | *Helicobacter* | 0.26 | ± | 0.02 | 0.56 | ± | 0.06 | 2.17 | 0.001 |
| Clostridium cl. XVII | *Coprobacillus catenoformis et rel.* | 0.89 | ± | 0.09 | 1.22 | ± | 0.12 | 1.37 | 0.048 |
| Clostridium cl. IV | *Anaerotruncus* | 1.53 | ± | 0.06 | 1.28 | ± | 0.11 | -1.19 | 0.030 |
| Bacilli | *L. paracasei et rel.* | 0.83 | ± | 0.06 | 0.60 | ± | 0.03 | -1.38 | 0.011 |
| Actinobacteria | *Bifidobacterium* | 0.49 | ± | 0.03 | 0.34 | ± | 0.04 | -1.45 | 0.011 |
| Clostridium cl. XIVa | *Butyrivibrio crossotus et rel.* | 1.42 | ± | 0.13 | 0.92 | ± | 0.13 | -1.54 | 0.030 |
| Clostridium cl. XIVa | *Eub. plexicaudatum et rel.* | 4.10 | ± | 0.31 | 2.36 | ± | 0.39 | -1.74 | 0.011 |
| Proteobacteria | *Labrys methylaminiphilus et rel.* | 0.51 | ± | 0.04 | 0.29 | ± | 0.09 | -1.76 | 0.030 |
| Actinobacteria | *Eggerthella* | 1.59 | ± | 0.28 | 0.88 | ± | 0.06 | -1.80 | 0.009 |
| TM7 | *Un. TM7* | 0.88 | ± | 0.07 | 0.48 | ± | 0.09 | -1.83 | 0.011 |
| Mollicutes | *Solobacterium moorei et rel.* | 0.67 | ± | 0.11 | 0.35 | ± | 0.02 | -1.91 | 0.006 |
| Clostridium cl. IV | *Dorea* | 2.33 | ± | 0.24 | 1.04 | ± | 0.24 | -2.24 | 0.011 |
| Clostridium cl. XIVa | *Lachnospira pectinoschiza et rel.* | 0.70 | ± | 0.06 | 0.29 | ± | 0.09 | -2.39 | 0.011 |
| Clostridium cl. XIVa | *Uncl. Clostridium cl XIVa* | 11.73 | ± | 0.95 | 4.85 | ± | 0.71 | -2.42 | 0.001 |
| Clostridium cl. XIVa | *Bryantella* | 2.20 | ± | 0.30 | 0.85 | ± | 0.16 | -2.59 | 0.002 |
| Clostridium cl. IV | *Sporobacter termitidis et rel.* | 8.47 | ± | 0.73 | 3.22 | ± | 0.52 | -2.63 | 0.002 |
| Clostridium cl. II | *Cl. lactifermentans et rel.* | 0.35 | ± | 0.06 | 0.12 | ± | 0.04 | -3.07 | 0.011 |
| Clostridium cl. XIVa | *Lachnobacillus bovis et rel.* | 0.94 | ± | 0.09 | 0.22 | ± | 0.03 | -4.32 | 0.001 |
| Clostridium cl. XVI | *Allobaculum* | 1.64 | ± | 0.37 | 0.25 | ± | 0.03 | -6.54 | 0.001 |
| Bacilli | *L. salivarius et rel.* | 0.05 | ± | 0.02 | 0.01 | ± | 0.00 | -6.65 | 0.001 |
| Bacilli | *Streptococcus intermedius et rel.* | 0.04 | ± | 0.01 | 0.01 | ± | 0.00 | -7.24 | 0.002 |
| Clostridium cl. IX | *Peptococcus niger et rel.* | 0.28 | ± | 0.04 | 0.02 | ± | 0.00 | -12.13 | 0.001 |
| Bacilli | *L. plantarum et rel.* | 0.11 | ± | 0.05 | 0.00 | ± | 0.00 | -12.99 | 0.023 |
| Mollicutes | *Acholeplasma* | 0.18 | ± | 0.04 | 0.01 | ± | 0.00 | -15.66 | 0.001 |
| Actinobacteria | *Olsenella et rel.* | 0.31 | ± | 0.11 | 0.00 | ± | 0.00 | -169.08 | 0.001 |

Data are given as % of total bacteria (see Table 3) and are represented as mean ± SEM (n=8). q-value is based on Mann-Whitney followed by Benjamini-Hochberg correction for multiple testing.
