# Supplementary material for: Dietary Heme Alters Microbiota and Mucosa of Mouse Colon without Functional Changes in Host-Microbe Cross-Talk
Source: PLoS One. 2012 Dec 11;7(12):e49868. doi: 10.1371/journal.pone.0049868 (PMC3519815; doi:10.1371/journal.pone.0049868)
Supplement: Materials and Methods S1 — (DOC) [file pone.0049868.s004.doc]

**Supporting information for:**

# Dietary heme alters microbiota and mucosa of mouse colon without functional changes in host-microbe cross-talk

Noortje IJssennagger, Muriel Derrien, Gerdien M. van Doorn, Anneke Rijnierse, Bartholomeus van den Bogert, Michael Müller, Jan Dekker, Michiel Kleerebezem, and Roelof van der Meer

**Supplementary Materials and Methods**

**Histology** Haematoxylin and eosin (H&E) staining was performed on colon tissue of all animals to investigate tissue morphology. Furthermore, an Alcian blue/PAS staining (staining neutral and acidic mucins), and a High Iron Diamine staining (HID, staining sulfated and carboxylated mucins), were performed. For both stainings, colon sections were deparaffinized and rehydrated in a series of graded alcohols. For the Alcian blue/PAS staining, the sections were incubated with Alcian blue (pH=2.5) for 30 min, then with periodic acid for 10 min and subsequently in Schiff’s reagent for 10 min. For the HID staining, deparaffinized sections were incubated overnight in diamine solution in a dark and humid atmosphere. Then the sections were incubated with Alcian blue (pH=2.5) for 30 min. Between all incubations, sections were rinsed with water. Before mounting, sections were dehydrated in a series of graded alcohols and xylene. For the detection of Alkaline Phosphatase, colon tissue slides were deparaffinized and incubated with the alkaline-dye mixture (Alkaline phosphatase kit 85L2-1KT Sigma Aldrich) for 90 min at 37°C. Slides were rinsed with water and mounted.

**Thiobarbituric Acid Reactive Substances (TBARS) assay.** The assay used determines lipid peroxidation by quantifying the concentration of malondialdehyde (MDA) in fecal water . Fecal water was diluted 4-fold with double-distilled water. To 100 µl of this dilution, 100 µl of 8.1% SDS and 1 ml of 0.11 mol/L 2,6-di-tert-butyl-p-cresol, 0.5% TBA in 10% acetic acid (pH 3.5) was added. To correct for background, TBA was omitted from the assay. TBARS were extracted, after heating for 75 minutes at 82°C, with 1.2 ml n-butanol. The absorbance of the extracts was measured at 540 nm. The amount of TBARS was calculated as MDA equivalents using 1,1,3,3,-tetramethoxypropane as standard.

**Bacterial DNA extraction.** Approximately 0.1 g was used for mechanical and chemical lysis using 0.5 ml buffer (500 mM NaCl, 50 mM Tris-HCl (pH 8), 50 mM EDTA, 4% SDS) and 0.25 g of 0.1 mm zirconia beads and 3 mm glass beads. Nucleic acids were precipitated by addition of 130 μl, 10 M ammonium acetate, using one volume of isopropanol. Subsequently, DNA pellets were washed with 70% ethanol. Further purification of DNA was performed using the QiaAmp DNA Mini Stool Kit (Qiagen, Hilden, Germany). Finally, DNA was dissolved in 200 μl Tris/EDTA buffer and its purity and quantity were checked spectrophotometrically (ND-1000, nanoDrop technologies, Wilmington, USA).

**Quantitative qPCR.** qPCR was performed using an IQ5 Cycler apparatus (Bio-Rad, Veenendaal, The Netherlands). Reactions were performed in triplicate in a single run. Samples were analyzed in a 25 μl reaction mixture consisting of 12.5 μl Bio-Rad master mix SYBR Green (50 mM KCl, 20 mM Tris-HCl, pH 8.4, 0.2 mM of each dNTP, 0.625 U i*Taq* DNA polymerase, 3 mM MgCl_2_, 10 nM fluorescein), 0.1 µM of each primer and 5 μl of template faecal DNA diluted 100- or 1000-fold. Standard curves of 16 S rRNA or specific gene PCR product from pure culture were created using serial 10-fold dilution of the purified PCR product. Standards included *Lactobacillus casei* (total bacteria), *Desulfovibrio* sp. (sulfate reducer) and *Pseudomonas aeruginosa* (nitrate reducer). The following qPCR conditions were used: 95°C for 10 min, followed by 35 cycles of denaturation at 95°C for 15 sec, annealing temperature of 60°C for 20 sec, extension at 72°C for 30 sec and a final extension step at 72°C for 5 min. A melting curve was determined at the end of each run to verify the specificity of the PCR amplicons. Data analysis was performed using the Bio-Rad software.

**MITChip procedure.** The full length 16S rRNA gene was amplified using primers T7prom-Bact-27-forward and Uni-1492-reverse . The PCR products were transcribed into RNA and the resulting RNA was labeled with Cy3 and Cy5 prior to fragmentation and hybridization to the array. Arrays were scanned with Agilent DNA Microarray Scanner to collect fluorescence data, which were then extracted from the generated images by using the Agilent Extraction Software 9.1. The array normalization was performed as described earlier . In summary, each scanner channel of each of the arrays was first spatially normalized with polynomial regression, after which the outliers in each set of probes were detected with a chi-squared test and discarded. The logarithmic intensities from the spatially normalized, reproducible, and sample-wise quantile-normalized hybridizations were then averaged to a single value for each probe and each sample in the data set.

**References**

1. Ohkawa H, Ohishi N, Yagi K (1979) Assay for lipid peroxides in animal tissues by thiobarbituric acid reaction. Analytical biochemistry 95: 351-358.

2. Frank JA, Reich CI, Sharma S, Weisbaum JS, Wilson BA, et al. (2008) Critical Evaluation of Two Primers Commonly Used for Amplification of Bacterial 16S rRNA Genes. Appl Environ Microbiol 74: 2461-2470.

3. Rajilic-Stojanovic M, Heilig H, Molenaar D, Kajander K, Surakka A, et al. (2009) Development and application of the human intestinal tract chip, a phylogenetic microarray: analysis of universally conserved phylotypes in the abundant microbiota of young and elderly adults. Environmental Microbiology 11: 1736-1751.

4. Suzuki MT, Taylor LT, DeLong EF (2000) Quantitative Analysis of Small-Subunit rRNA Genes in Mixed Microbial Populations via 5'-Nuclease Assays. Appl Environ Microbiol 66: 4605-4614.

5. Ben-Dov E, Brenner A, Kushmaro A (2007) Quantification of Sulfate-reducing Bacteria in Industrial Wastewater, by Real-time Polymerase Chain Reaction (PCR) Using dsrA and apsA Genes. Microbial Ecology 54: 439-451.

6. Bru D, Sarr A, Philippot L (2007) Relative Abundances of Proteobacterial Membrane-Bound and Periplasmic Nitrate Reductases in Selected Environments. Appl Environ Microbiol 73: 5971-5974.
